# Waiting lists in health care: multiple perspectives

**DOI:** 10.1590/0102-311XEN098925

**Published:** 2025-06-20

**Authors:** Everton Nunes da Silva

**Affiliations:** 1 Universidade de Brasília, Brasília, Brasil.

In the current issue, CSP published an essay on issues related to waiting lists in specialized outpatient care written by Giannotti et al. ^1^. In the study, the authors address several issues relevant to understanding and problematizing this topic in health systems, focusing particularly on the Brazilian Unified National Health System (SUS, acronym in Portuguese). Among these issues, conceptual aspects, the dimensioning of this problem in health care, the available regulatory devices and reflections on the transparency of waiting lists stand out. In the context of specialized outpatient care, waiting lists mainly cover consultations, exams and elective procedures. 

Waiting too long to be served can create substantial problems for people and the health system ^2^. In individual terms, late diagnosis due to long delays in accessing specialist consultations and tests can reduce the chances of a more favorable prognosis, impacting survival and quality of life. As for the health system, the worsening health status of individuals reduces therapeutic options and increases treatment costs, requiring budget increases that are not always feasible. Hence why monitoring waiting lists is essential to ensure SUS integrality and sustainability. 

Additionally, waiting lists can also signal weaknesses in the SUS coordination of health care, reflecting the fragmentation of care ^3^. Despite the progress made during the implementation of SUS, challenges remain in terms of continuity in medical care and the articulation of health services with different technological densities ^4^. This scenario can contribute to duplicate requests for tests and consultations, particularly when information systems do not allow medical records to be shared. It is therefore difficult to measure how much the waiting lists actually reflect the real health needs of the population served. At least two points provide theoretical and empirical support for this statement: the adoption of fee for service remuneration and the existence of asymmetric information (induced demand). 

Remuneration based on fee for service tend to stimulate the volume of production, unnecessarily and without adding value to diagnosis or user treatment ^5^. Such modus operandi is perpetuated because the income of providers (health professionals and facilities) depends on the quantity produced, following a “the more, the better” logic. In other words, the concern with quantity can override the quality of care. This remuneration model is predominant in the Brazilian health system, especially in the SUS. Such adverse economic incentive generates inefficiencies and burdens on health systems, particularly those with fragmented systems and limited budgets ^6^. 

Supplier-induced demand also generates unnecessary consumption and, consequently, inefficiencies for health systems ^7^. Its origin lies in the asymmetric information between the economic agents, in this case the health professionals, and the patient when they define the procedures necessary to establish a diagnosis or treatment. Supplier-induced demand occurs when the patient’s decision on the number of procedures is less than that proposed by the healthcare professional, if the former had the same medical knowledge as the latter ^8^. Thus, some individuals on waiting lists for appointments, exams and elective procedures may not necessarily need these resources. Supplier-induced demand ends up exacerbating the problem of long waiting times.

One way to mitigate the problems related to fee for service and supplier-induced demand is to implement clinical practice guidelines. These signal the health needs for each disease or health condition based on systematic and transparent processes of summarizing the best available scientific evidence ^9^. In addition to the quality and strength of recommendation in said guidelines, it is important to establish implementation strategies to increase the chances of evidence-based practices being effectively used in clinical care ^10^. A study conducted in Poland ^11^ showed that the implementation of an education intervention for the use of evidence-based practices reduced the number of unnecessary medical procedures by 20% and the costs of outpatient procedures by 18% after two years of the intervention, while maintaining relatively high patient satisfaction with medical treatment . 

Another point highlighted by Giannotti et al. ^1^ refers to absenteeism on waiting lists, defined as not showing up for scheduled appointments, exams or procedures without any apparent justification. Absenteeism is a global phenomenon which reaches high proportions in Brazil, accounting for 25% of cases on the SUS waiting list ^12^. In this context, a promising field of research has opened up with the use of artificial intelligence and machine learning applied to the scheduling of health procedures. A recent literature review pointed to heterogeneous and uncertain results regarding the potential of these approaches in predicting possible absenteeism in healthcare services ^13^. Additionally, mastering these approaches will require a high level of technological capacity on the part of health services which could increase inequalities in the SUS.

Giannotti et al. ^1^ stimulate discussion of various points of view and methodological approaches, both in investigating the causes of excessive waiting times on lists and in proposing strategies to overcome this problem. Regardless of the path to be followed, studies that address waiting lists in the SUS have a high potential to contribute both in improving management and advancing knowledge.
