# Supplementary material for: Waiting lists in health care: multiple perspectives
Source: Cad Saude Publica. 2025 Jun 20;41(6):e00098925. doi: 10.1590/0102-311XEN098925 (PMC12185043; doi:10.1590/0102-311XEN098925)
Supplement: Translation

# Translation: Listas de espera em saúde: múltiplos olhares

**DOI:** 10.1590/0102-311XPT098925

**Authors:** Everton Nunes da Silva

**Affiliations:** 1 Universidade de Brasília, Brasília, Brasil.

No fascículo atual, CSP publicou um ensaio sobre questões relacionadas às listas de espera na atenção ambulatorial especializada, de autoria de Giannotti et al. ^1^. No estudo, os autores abordam várias questões relevantes para o entendimento e problematização dessa temática nos sistemas de saúde, com especial foco no Sistema Único de Saúde (SUS). Entre essas questões, destacam-se os aspectos conceituais, o dimensionamento deste problema na saúde, os dispositivos regulatórios disponíveis e as reflexões sobre a transparência das listas de espera. No âmbito da atenção ambulatorial especializada, as listas de espera abrangem principalmente consultas, exames e procedimentos eletivos. 

Tempos demasiados na espera por cuidados de saúde podem gerar problemas substanciais às pessoas e ao sistema de saúde ^2^. Em termos individuais, o diagnóstico tardio devido a longos períodos para ter acesso a consultas de especialidade e exames pode reduzir as chances de ter um prognóstico mais favorável, impactando na sobrevida e na qualidade de vida dos indivíduos. Em relação ao sistema de saúde, o agravamento do estado de saúde dos indivíduos reduz as opções terapêuticas e aumenta os custos do tratamento, necessitando de incrementos orçamentários que nem sempre são factíveis. Assim, monitorar as listas de espera é essencial para garantir a integralidade e sustentabilidade do SUS. 

Adicionalmente, as listas de espera também podem sinalizar fragilidades na coordenação da atenção à saúde no SUS, refletindo a fragmentação do cuidado ^3^. A despeito dos avanços conquistados ao longo da implementação do SUS, ainda persistem desafios de continuidade na assistência médica e na articulação dos serviços de saúde com diferentes densidades tecnológicas ^4^. Esse contexto pode contribuir para a duplicidade de solicitações de exames e consultas, particularmente quando os sistemas de informação não permitem o compartilhamento de prontuários médicos. Dessa forma, é difícil mensurar o quanto realmente as listas de espera refletem as reais necessidades de saúde da população atendida. Pelo menos dois pontos dão sustentação teórico-empírica a essa afirmação: a adoção de sistemas de remuneração baseados em quantidade (*fee for service*) e a existência de assimetria de informação (demanda induzida). 

Os sistemas de remuneração baseados em quantidade de procedimentos tendem a estimular o volume de produção de forma desnecessária e sem adicionar valor ao diagnóstico ou tratamento dos usuários ^5^. Este *modus operandi* se perpetua porque os rendimentos dos provedores (profissionais e estabelecimentos de saúde) dependem da quantidade produzida, seguindo a lógica de “quanto mais, melhor”. Em outros termos, a preocupação com a quantidade pode se sobrepor à qualidade do cuidado. Este modelo de remuneração é predominante no sistema de saúde brasileiro, especialmente no SUS. Este incentivo econômico adverso gera ineficiências e sobrecarga aos sistemas de saúde, particularmente naqueles com sistema fragmentados e com orçamentos limitados ^6^. 

A demanda induzida também gera consumo desnecessário e, consequentemente, ineficiências aos sistemas de saúde ^7^. Sua origem reside na assimetria de informação entre os agentes econômicos que, neste caso, seriam o profissional de saúde e o paciente quando estes definem os procedimentos necessários para estabelecer o diagnóstico ou tratamento. A demanda induzida ocorreria quando a decisão do paciente sobre a quantidade de procedimentos fosse menor do que a proposta pelo profissional da saúde, caso o paciente tivesse o mesmo conhecimento em medicina que o profissional de saúde ^8^. Assim, pode haver pessoas nas listas de espera por consultas, exames e procedimentos eletivos que não necessariamente precisem desses recursos. A presença de demanda induzida acaba exacerbando o problema de longos períodos de espera. 

Uma forma de mitigar os problemas relacionados ao *fee for service* e a demanda induzida é a implementação de guias de práticas clínicas. Estes sinalizam as necessidades de saúde para cada doença ou condição de saúde, baseados em processos sistemáticos e transparentes de sumarização das melhores evidências científicas disponíveis ^9^. Além da qualidade e da força de recomendação dos guias, é importante estabelecer estratégias de implementação no sentido de aumentar as chances de que as práticas baseadas em evidências sejam efetivamente usadas nos atendimentos clínicos ^10^. Estudo conduzido na Polônia ^11^ mostrou que a implementação de uma intervenção de educação para uso de práticas baseadas em evidências reduziu em 20% o número de procedimentos médicos desnecessários e em 18% os custos de procedimentos ambulatoriais após dois anos da intervenção, mantendo a satisfação dos pacientes relativamente alta com o tratamento médico. 

Outro ponto ressaltado pelos autores ^1^ refere-se ao absenteísmo nas listas de espera, definido como o não comparecimento às consultas, exames ou procedimentos agendados, sem justificativa aparente. O absenteísmo é um fenômeno global, mas no Brasil ele atinge proporções elevadas, representando 25% dos casos em lista de espera no SUS ^12^. Nesse contexto, tem-se aberto um campo promissor de pesquisa com o uso de inteligência artificial e aprendizado de máquina (*machine learning*) aplicados ao agendamento de procedimentos em saúde. Recente revisão de literatura apontou resultados heterogêneos e incertos sobre o potencial dessas abordagens na predição de eventuais faltosos nos atendimentos de saúde ^13^. Além disso, o domínio dessas abordagens demandará uma capacidade tecnológica elevada dos serviços de saúde, o que pode ampliar as desigualdades no SUS.

O estudo de Giannotti et al. ^1^ estimula a discussão de vários pontos de vistas e abordagens metodológicas, seja na investigação das causas dos excessivos tempos de espera nas listas, seja na proposição de estratégias para contornar esse problema. Independentemente do caminho a trilhar, estudos que abordem as listas de espera no SUS têm alto potencial de contribuição tanto em termos de aprimoramento da gestão quanto de avanço no conhecimento.

# Translation: Listas de espera en salud: múltiples miradas

**DOI:** 10.1590/0102-311XES098925

**Authors:** Everton Nunes da Silva

**Affiliations:** 1 Universidade de Brasília, Brasília, Brasil.

En el número actual, CSP ha publicado el ensayo de Giannotti et al. ^1^ sobre las listas de espera en la atención ambulatoria especializada. En el estudio, los autores abordan una problemática que permite comprender y problematizar esta cuestión en los sistemas de salud, bajo una mirada especial al Sistema Único de Salud brasileño (SUS). Entre la problemática abordada destacan los aspectos conceptuales, el dimensionamiento de esta cuestión en salud, las disposiciones normativas disponibles y las reflexiones sobre la transparencia de las listas de espera. En la atención ambulatoria especializada, las listas de espera cubren, principalmente, consultas, exámenes y procedimientos electivos. 

Demasiado tiempo de espera para la atención médica puede generar problemas sustanciales a las personas y al sistema de salud ^2^. En ámbito individual, el diagnóstico tardío debido a largos períodos para acceder a consultas y pruebas especializadas puede reducir las probabilidades de tener un pronóstico favorable, lo que afecta la supervivencia y la calidad de vida de las personas. Respecto al sistema de salud, el empeoramiento del estado de salud de las personas reduce las opciones terapéuticas y aumenta los costos de tratamiento, lo que requiere aumentos presupuestarios que, muchas veces, no son factibles. Por lo tanto, el seguimiento de las listas de espera es esencial para garantizar la integridad y la sostenibilidad del SUS. 

Además, las listas de espera también pueden indicar debilidades en la coordinación de la atención médica en el SUS, lo que refleja la fragmentación de la atención ^3^. A pesar de los avances durante la implementación de este sistema, aún existen desafíos en la continuidad de la atención médica y en la articulación de los servicios de salud con diferentes tecnologías ^4^. Este contexto puede influir en la duplicación de solicitudes de exámenes y consultas, sobre todo cuando los sistemas de información no permiten compartir los registros médicos. Y así es difícil medir cuánto las listas de espera reflejan las necesidades reales de salud de la población atendida. Al menos dos puntos proporcionan soporte teórico y empírico a esta afirmación: la adopción de sistemas de remuneración basados en la cantidad (*fee for service*) y la existencia de asimetría de información (demanda inducida). 

Los sistemas de remuneración basados en el número de procedimientos tienden a estimular el volumen de producción innecesariamente y sin agregar valor al diagnóstico o tratamiento de los usuarios ^5^. Este modus operandi continúa porque los ingresos de los proveedores (profesionales y centros de salud) dependen de la cantidad producida con base en la lógica de “cuanto más, mejor”. En otras palabras, la preocupación por la cantidad puede superponerse a la calidad de la atención. Este modelo de remuneración es predominante en el sistema de salud brasileño, en particular en el SUS. El incentivo económico adverso genera ineficiencias y sobrecarga los sistemas de salud, principalmente en aquellos con sistemas fragmentados y presupuestos limitados ^6^. 

La demanda inducida también genera consumos innecesarios y, en consecuencia, ineficiencias en los sistemas de salud ^7^. Su origen radica en la asimetría de información entre agentes económicos, que, en este caso, son el profesional sanitario, y el paciente al definir los procedimientos necesarios para establecer el diagnóstico o tratamiento. La demanda inducida se produciría cuando la decisión del paciente sobre el número de procedimientos es menor que la propuesta por el profesional de la salud, si el paciente tuviera los mismos conocimientos en medicina que el profesional de la salud ^8^. Por lo tanto, es probable que haya personas en listas de espera para consultas, exámenes y procedimientos electivos que no necesariamente los necesitan. La presencia de demanda inducida termina por exacerbar el problema de largos períodos de espera. 

Una forma de mitigar los problemas relacionados con el *fee for service* y la demanda inducida es la implementación de guías de práctica clínica. Estas orientaciones indican las necesidades de salud para cada enfermedad o condición de salud con base en procesos sistemáticos y transparentes para resumir la mejor evidencia científica disponible ^9^. Más allá de la calidad y la solidez de la recomendación de las guías, es importante establecer estrategias de implementación para aumentar las posibilidades de que las prácticas basadas en evidencia se utilicen efectivamente en la atención clínica ^10^. Un estudio realizado en Polonia ^11^ reveló que la implementación de una intervención educativa para el uso de prácticas basadas en evidencia redujo un 20% en los procedimientos médicos innecesarios y un 18% en los costos de los procedimientos ambulatorios tras dos años de la intervención, lo cual permitió mantener una alta satisfacción de los pacientes con el tratamiento médico. 

Otro punto destacado por Giannotti et al. ^1^ se refiere al absentismo en las listas de espera, es decir, la no asistencia a consultas, exámenes o procedimientos programados, sin justificación. Aunque el absentismo es un fenómeno global, en Brasil alcanza altos niveles, con el 25% de los casos en lista de espera en el SUS ^12^. Este contexto ha abierto un campo de investigación prometedor a partir del uso de la inteligencia artificial y del aprendizaje automático (*machine learning*) aplicado a la programación de procedimientos de salud. Una revisión reciente de la literatura señaló resultados heterogéneos e inciertos sobre el potencial de estos enfoques para predecir posibles ausencias en la atención médica ^13^. Además, el dominio de estos enfoques requerirá una alta capacidad tecnológica de los servicios de salud, lo que puede aumentar las desigualdades en el SUS.

El estudio de Giannotti et al. ^1^ incita al debate sobre diversos puntos de vista y enfoques metodológicos, ya sea en la investigación de las causas del tiempo de espera excesivo en las listas o en la propuesta de estrategias para superar este problema. Sin importar el camino que recorrer, los estudios sobre las listas de espera en el SUS tienen mucho por contribuir a mejorar la gestión y avanzar el conocimiento.
